# Review of natural compounds for potential psoriasis treatment

**DOI:** 10.1007/s10787-023-01178-0

**Published:** 2023-03-30

**Authors:** Omali Y. Elkhawaga, Mohamed M. Ellety, Sheref O. Mofty, Mohamed S. Ghanem, Abdallah O. Mohamed

**Affiliations:** Biochemistry Division, Chemistry Department, Faculty of Science, Mansoura University, Mansoura, 35516 Egypt

**Keywords:** Psoriasis, Autoimmune, Plant, Natural, Dietary, Phytochemical

## Abstract

Psoriasis represents an immune-mediated disease with an unclear cause that’s marked by inflammation triggered by dysfunction in the immune system, which results in inflammation in various parts of the skin. There could be obvious symptoms, such as elevated plaques; these plaques may appear differently depending on the type of skin. This disease can cause inflammation in the elbows, lower back, scalp, knees, or other regions of the body. It can begin at any age, although it most commonly affects individuals between the ages of 50 and 60. Specific cells (such as T cells) have been observed to play an obvious role in the pathogenesis of psoriasis, in addition to specific immunological molecules such as TNF-, IL-12, IL-23, IL-17, and other molecules that can aid in the pathogenesis of psoriasis. So, during the past two decades, biologists have created chemical drugs that target these cells or molecules and therefore prevent the disease from occurring. Alefacept, efalizumab, Adalimumab, Ustekinumab, and Secukinumab are a few examples of chemical drugs. It was discovered that these chemical drugs have long-term side effects that can cause defects in the patient's body, such as the development of the rare but life-threatening disorder progressive multifocal leukoencephalopathy (PCL). Its rapidly progressive infection of the central nervous system caused by the JC virus and other drugs may cause increased production of neutralising anti-drug antibodies (ADA) and the risk of infusion reactions like pruritus, flushing, hypertension, headache, and rash. So, our context intends to talk in our review about natural products or plants that may have therapeutic characteristics for this disease and may have few or no side effects on the patient's body.

## Introduction

Psoriasis represents an autoimmune, persistent inflammatory disease that affects the skin and has a powerful hereditary component. It is distinguished by persistent inflammation, which results in uncontrolled keratinocyte growth and differentiation (Pathogenesis 2019). In accordance with the International Psoriasis Day Collaboration, psoriasis affects 125 million individuals globally, or around 2–3% of the population. According to research, psoriatic arthritis affects 10–30% of psoriasis sufferers. Psoriasis has a detrimental influence on sufferers' quality of life. Even though psoriasis` pathophysiology remains unknown, it is thought to be a T-cell-triggered disorder because of the presence of T-helper cells inside the psoriatic state (Pathogenesis 2019; Theses and Devera 2020; Singhvi et al. 2020). Chronic plaque psoriasis (psoriasis vulgaris), inverse psoriasis, guttate psoriasis, Pustular psoriasis and erythrodermic psoriasis are the two clinical classifications of psoriasis. Chronic psoriasis vulgaris accounts for 80–90% of psoriasis patients. Psoriasis vulgaris can be distinguished by erythematous, well-defined plaques coated with silvery scales. Moreover, it can affect limb extensor surfaces, scalp, and trunk. Inverted psoriasis is characterised by somewhat erosive erythematous patches and plaques in the folds and vaginal regions. This condition is a mix of psoriasis and inflamed arthritis that affects the joints and spine. This happens when there are no particular antibodies in the blood or rheumatoid factor. Because of societal shame and marginalisation, psoriasis patients suffer poor mental and psychological health. Anxiety, sadness, and suicide thoughts are more common in these people (Napolitano, et al. 2016). The major signs of psoriasis appear as itchiness and reddish, flaky skin areas coated in silver scales. Other symptoms include tiny scale areas, cracked skin, itching, pain, swollen or corroded nails, inflammatory and genital sores, stiff joints, and extreme dandruff on the scalp (Global report 2022). Psoriasis is a complex disease influenced by genetic, environmental, and immunological factors. Modifying variables include obesity, illness, trauma, and a lack of active Vitamin D3.

## Pathogenesis

Psoriasis represents a common inflammatory skin condition with a complex pathophysiology that includes a hereditary element, immune dysfunction, and environmental exposures. It is linked to a variety of complications, including psoriatic arthritis, cardiac disease, metabolic syndrome, and obesity. Obesity appears to be a significant predictor for incident psoriasis, aggravates established psoriasis, and may improve the intensity of psoriasis in individuals. (Howell et al. 2019).

It is a genetic skin disease mediated by the immune system. The pathomechanisms at work involve complex communication between the immune system's innate and adaptive systems. T cells communicate with dendritic cells, keratinocytes, and macrophages via cytokines secreted by these cells. Streptococcal infection, smoking, stress, obesity, and alcohol consumption are all examples of environmental triggers (Kamiya et al. 2019).

Psoriasis is linked to a number of complications, including cardiovascular disease, cancer, and depression. For mild to severe psoriasis, corticosteroids, vitamin D mimics, and tazarotene were effective therapies. This illness can indeed be caused by a variety of microorganisms, including in psoriatic skin, where there was a rise in Corynebacterium, Propionibacterium, Streptococcus, and Staphylococcus (Thomas et al. 2021). Nevertheless, in another study, Staphylococci were considerably lower in lesional skin in comparison with healthy controls was a rise in Corynebacterium, Propionibacterium, Streptococcus, and Staphylococcus (Thomas et al. 2021). Nevertheless, in another study, Staphylococci were considerably lower in lesional skin in comparison with healthy controls. Psoriasis has been linked to fungi, including Malassezia as well as *Candida albicans*, as well as viruses like the human papillomavirus. Malassezia has been identified as the main prevalent fungus in both psoriatic and healthy skin. It has been discovered that disruptions in the adaptive and innate cutaneous immunogenicity are responsible for the formation and maintenance of psoriatic inflammation. Th1 cytokines, including c-interferon (IFN-c), tumour necrosis factor-a (TNF-a), and interleukin (IL)-12, were shown to be raised in psoriatic lesions, but Th2 cytokines (IL-4, IL-5, and IL-10) were not (Blauvelt and Chiricozzi 2018).

In psoriasis vulgaris areas, the bulk of epidermal T cells may generate type 1 cytokines, including INF-c, IL-2, and TNF-a, identifying cytotoxic T-lymphocytes (TC1) as well as TH1 effector populations. Psoriasis was identified as a Th1-type illness based on these findings. However, neither IFN-c nor TNF-a stimulate keratinocyte growth (Spa 2008).

**Fig. 1:**
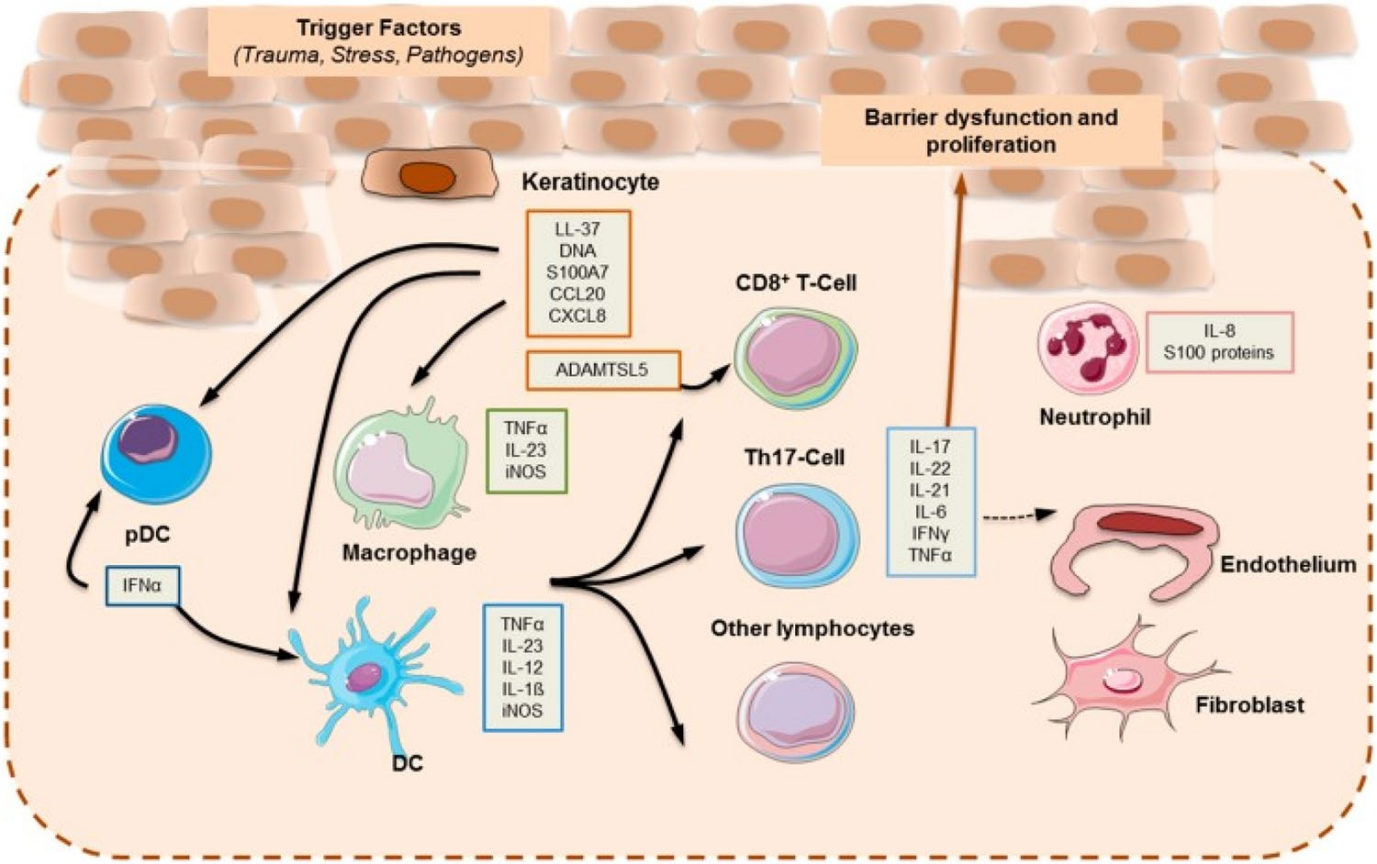
Basic pathogenesis of psoriasis

Interleukin-6 (IL-6) and other pro-inflammatory cytokines can activate the hypothalamus-pituitary axis, which has been linked to hypertension, obesity, and insulin resistance. IL-6 can also stimulate the production of C-reactive protein in hepatocytes and, in conjunction with TNF-, alter insulin sensitivity via the insulin signalling system (Liang et al. 2018). The formation of plaque in psoriasis is not isolated to inflammation at the epidermal layer; it is influenced by the keratinocyte’s interaction with many other types of cells (adaptive and innate immune cells, vasculature) across the dermis layer of skin. In certain cases, natural immune system activation caused by intrinsic danger signals or cytokines intermixes with autoinflammatory persistence, whereas in others, T cell-driven autoimmune responses occur. Thus, psoriasis exhibits disease characteristics on an auto-inflammatory basis, with both pathways overlapping and even amplifying each other (Meglio et al. 2014). The aetiology of psoriasis may be divided into two phases: the initiation phase, which may be induced by trauma (the Koebner phenomenon), infections, or medicines; and the maintenance phase, which is defined by continuous clinical development. Psoriasis pathogens originate once keratinocytes are exposed to an external stimulus (infection/stress/obesity/genetically), and as a result, keratinocytes secrete antimicrobial peptides (AMPs) that are overexpressed in psoriatic skin (Lai and Gallo 2009) (Fig. 1).

AMP are made up of twelve to fifty amino acids that help the host defend itself by destroying pathogens such as protozoa, bacteria, fungus, and certain viruses.It also has an impact on inflammatory responses by acting as an angiogenic factor, chemotactic mediator, and cell proliferation regulator. Several AMP, including -defensins, S100 proteins, and cathelicidin, are abundantly expressed in psoriatic lesions (Kiel 2004)**.**

The -defensins have been divided into six subtypes known as human neutrophil peptides, or 1-6HNP, of which HNP 1–3 are found in the scales of psoriatic lesions.-Defensins are divided into four subtypes known as 1–4 human -defensins (HD).

HD 1–2 were found to be highly expressed in psoriasis scales and are activated in keratinocytes by TNF- and/or IFN-c (Lande et al. 2014). Furthermore, IL-22 and IL-17A both cause HD 2. LL37, also known as cathelicidin, has been linked to the pathogenesis of psoriasis. Injured keratinocytes secrete LL37, which is then combined with self-genetic materials from those damaged cells to form complexes. LL37 represents one of the two T-cell autoantigens examined in psoriasis. Research revealed specific CD8+ and CD4+ T lymphocytes in two-thirds of individuals with mild to severe plaque psoriasis. LL37-specific T cells produce IFN-, while CD4+ T cells produce IL-22, IL-21, and IL-17. Specific T lymphocytes (LL37) could be detected in skin lesions and blood, and their presence correlates with disease activity (Morizane et al. 2012).

CD8+ T-cells stimulated by LL37 participate in autoantigen detection, epidermotropism, and Th17 cytokine release. The HLA-C*06:02-restricted autoantigen ADAMTSL5 was discovered to be recognised because of a CD8+ T-cell TCR that is autoreactive. This discovery identifies melanocytes as immune target cells. TLR9 in plasmacytoid DCs is activated by LL37 coupled to DNA (pDCs) (Arakawa, et al. 2015). The activation of pDC, characterised by the production of type I IFNs such as IFN- and IFN-, is essential for the formation of the psoriatic plaque. Type I IFN signalling promotes myeloid dendritic cell (mDC) phenotypic development and has been linked to Th1 and Th17 development and role, including the production of IFN- and IL-17, respectively (Morizane and Gallo 2011). LL37–DNA induces pDCs via LL37, and TLR9 bound to RNA induces pDCs through TLR7. Moreover, LL37–RNA complexes work on mDCs via TLR8.

Activated Th17 cells release an excess of IL-17 and TNF-a, which stimulate keratinocytes, increase hyperplasia of the epidermis, attract neutrophils, and cause the generation of antimicrobial peptides (AMP). IL-17 mRNA levels were shown to be elevated in psoriasis lesions but never in non-lesional tissue (Lowes et al. 2008).

IL-17 increased the levels of IL-8 and IL-6 in keratinocytes, which are cytokines that promote inflammation and aggravate psoriasis (Ye et al. 2001).

Furthermore, IL-17 is required for neutrophil preservation and recruitment. Th17 cells are also known to release IL-22, a participant in the IL-10 cytokine family (Wolk et al. 2006). Its expression can be elevated in the area of psoriatic lesions on the skin and decrease in connection with antipsoriatic treatment remission. These data imply that IL-22 stimulates keratinocyte development and plays a significant role in psoriasis pathogenesis.

TNF- (tumor necrosis factor) plays an important role in the pathophysiology of psoriasis (Jacobs and Harrison 1998). TNF- activates the nuclear factor (NF)-jB signalling pathway, which affects lymphocyte and keratinocyte survival, proliferation, and anti-apoptotic activities (Kagami et al. 2010).

TNF- stimulates Th17 to produce proinflammatory cytokines via the NF-jB pathway in psoriatic lesions, and blocking the NF-jB pathway reduces IL-17A production by CD4+ T cells. Both anti-TNF and CsA agents reduced the levels of IFN-c, IL-17A, IL-23p19, and (C–C motif) chemokine ligand 20 in psoriasis lesions, indicating that pro-inflammatory cytokines, particularly IL-17A, are involved in the development of psoriasis (Ghoreschi et al. 2010).

**Fig. 2:**
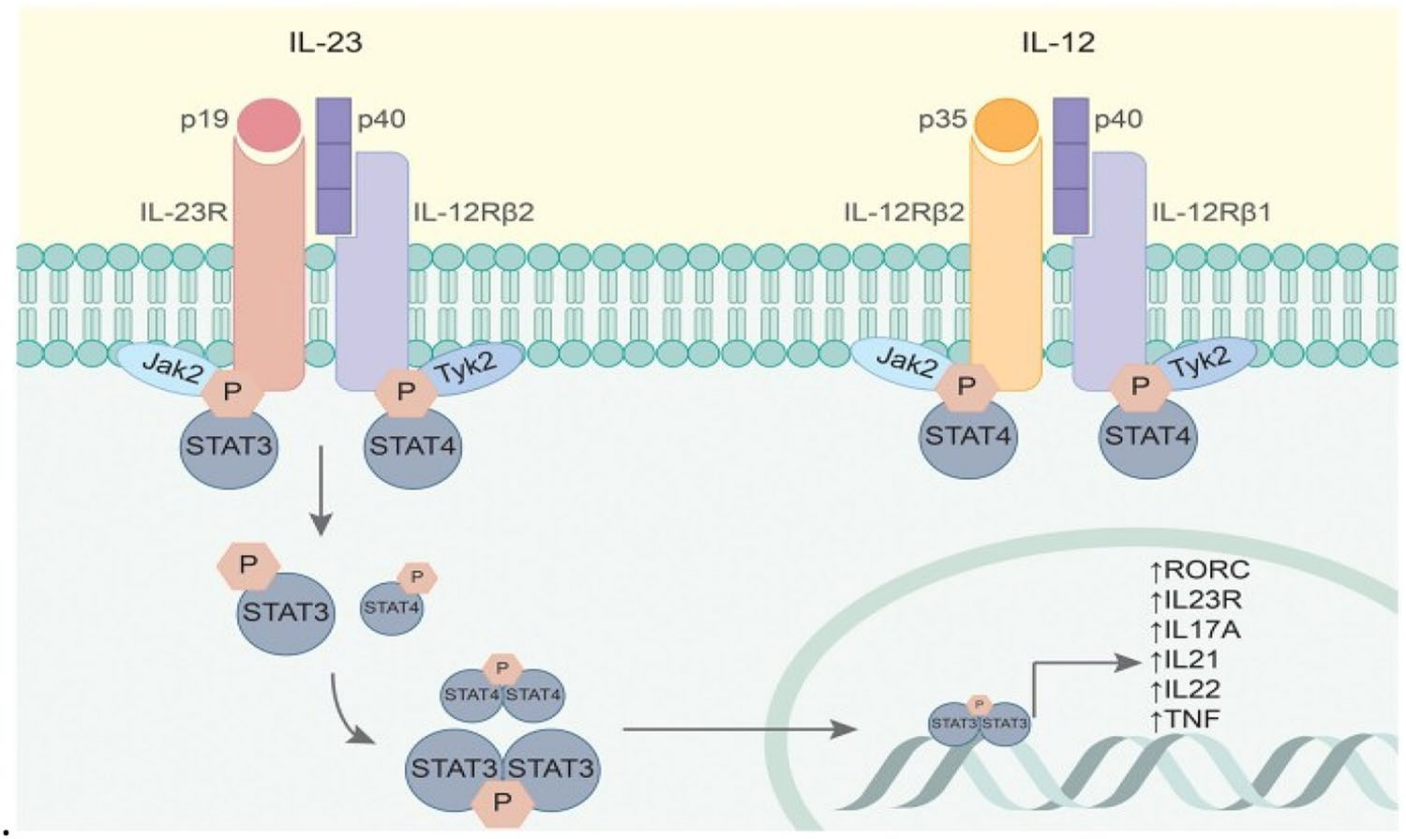
Schematic representation of IL-12 and IL-23, and their receptors and downstream signaling pathways

These data imply that the IL-17 family is involved in psoriasis. When IL-23 binds to the receptor complex of IL17, it phosphorylates signal transduction and the promoter of transcription (STAT)3 via Jak2, which is found inside the cytoplasmic domain of the subunit IL-23R. Phospho-STAT3 proteins create homodimers in the nucleus, where they boost the Th17 response by stimulating the production of cytokines such as IL-22, 17A, IL-17F, and IFN-c (Fig. 2).

The missing heritability research that has been linked with psoriasis genetic markers has fueled the epigenetic alterations search. Cytosine and guanine (CpG) methylation, silencing microRNA (miRNA), and long noncoding RNA (lncRNA) represent examples of epigenetic processes that affect gene expression without modifying the chromosomal sequence. More than 971 lncRNAs were identified as being differently expressed in psoriatic patches versus normal skin (Gupta et al. 2016).

## Psoriasis-related genes

### IL36RN

By 2011, IL36RN deficiency was discovered in a recessive variety of IL36RN, and familial GPP was identified as a causal gene for GPP (Johnston et al. 2012). The gene IL36RN encodes IL-36 receptor antagonists (IL36Ra), which inhibit the action of IL-36 (including IL-36a, IL-36b, and IL-36c) from the IL-1 family. Those subtypes are extensively expressed in affected lesions and seem to be important regulators for neutrophil chemokines such as CXCL1 and CXCL8 (Blumberg et al. 2007). The mouse homolog of IL36RN, Il1f5, has similarly been demonstrated to cause a skin condition in comparison with GPP in mice; stimulation of neutrophils in GPP may be caused by elevated IL-36 activity caused by IL36Ra malfunction.

### CARD14

Caspase recruiting member 14 (CARD14) or (CARMA2) is a scaffold protein that regulates NF-jB signalling via TNF receptor-associated factor 2 (TRAF2). Many mutations accompanied by function gains in CARD14, including p. Gly117Ser, were discovered in GPP (Setta-kaffetzi et al. 2014).

#### AP1S3

AP1S3 generates a component of AP-1, a transcription element that affects the expression of keratin in keratinocytes. GPP patients were found to have AP1S3 mutations and may stimulate CXCL8, IL-36, and IL-1b expression via NF-jB signalling malfunctions. It was discovered that the differentiation of specific keratins 1 and 10, which are mostly expressed inside the spinous layers, is reduced in the affected epidermis, whereas keratins 6 and 16 are enhanced. 55 Furthermore, in psoriatic skin, transglutaminase 1, keratin 17, and involucrin are raised, although profilagrin is reduced (Wright 1991).

## Natural sources of psoriasis treatment

### Omega-3 sources

A number of studies have explored the efficacy of dietary supplementation using fish oils (Article 2018), partly because fish oil contains high levels of LC-3 PUFAs and partly because of the theory that increased production of pro-inflammatory eicosanoids via ARA is important to the pathophysiology of psoriasis. In humans, -3 PUFAs perform a variety of activities, including restricting and healing inflammatory processes. They have been extensively researched for their capacity to reduce mortality and morbidity in people with cardiovascular disease (Hamilton et al. 1990). PUFAs have been used as a safe adjunct therapy in various skin diseases due to their anti-inflammatory and anti-chemotactic properties. Inhibition of inflammation by ALA and its derivatives is based on barrier function maintenance, stratum corneum maturation and differentiation, proinflammatory eicosanoid inhibition, lamellar body formation, cytokine suppression, and lipoxygenase inhibition (Ricketts et al. 2010).

Resolvens control neutrophil migration in both animals and humans, which may be one of the key anti-inflammatory effects of -3 FAs on psoriasis (Mccusker and Grant-kels 2010). Nevertheless, animal research has indicated the -3 PUFAs' therapeutic benefit for lesions resembling psoriasis, but human studies have yielded conflicting findings (Maurice et al. 1987). According to in vitro studies, adding fish oil to the diets of psoriasis patients results in an increase in plasma EPA-to-ARA ratio and platelets, as well as a significant decrease in LTB4 production by neutrophils (Soyland et al. 1993). Additional advantages of -3 PUFA administration in psoriasis patients include possible hypolipidemic consequences and the avoidance of insulin resistance and obesity. Because psoriasis sufferers are more likely to be overweight than the overall population (Watson 2013), reducing the inflammatory load associated with an elevation in the quantity of fatty tissue might undoubtedly help to treat psoriasis. This is due to increased levels of systemic circulating inflammatory cytokines like IL-6, TNF-, and adiponectin produced by visceral fat activating rapamycin complex 1 (mTORC1) signaling, which is important for controlling T-cell homeostasis as well as proinflammatory signalling of keratinocytes via the NF-B pathway (Timoszuk and Bielawska 2018).

A study was done to explore the effectiveness of petroleum ether that has been extracted from Annona squamosa seed (ASO) for use as an antipsoriatic drug (Bhoir et al. 2018). In mice, the extract, which is mostly composed of PUFAs (LA and OA), inhibits keratinocyte proliferation more effectively than the corticosteroid clobetasol propionate (CP). There was a reduction in inflammatory lesion cytokines (INF-, TNF-, IL6, IL17, and GM-CSF) after ASO administration, and no side effects were seen, indicating that further research into this new antipsoriatic cream for treatment in humans is warranted. PUFA supplementation might help with psoriasis therapy and comorbidity prevention. Even a slight improvement in psoriasis severity caused by PUFA may reduce the use of currently available medications, lowering the risk of adverse effects.

### Vitamin D

The vitamin D system is important for intracellular calcium and bone metabolism, although studies throughout the last 20 years have shown a wide variety of biological activities, including cell differentiation, suppression of cellular development, immunomodulation, and regulation of many other hormonal systems.

**Fig. 3:**
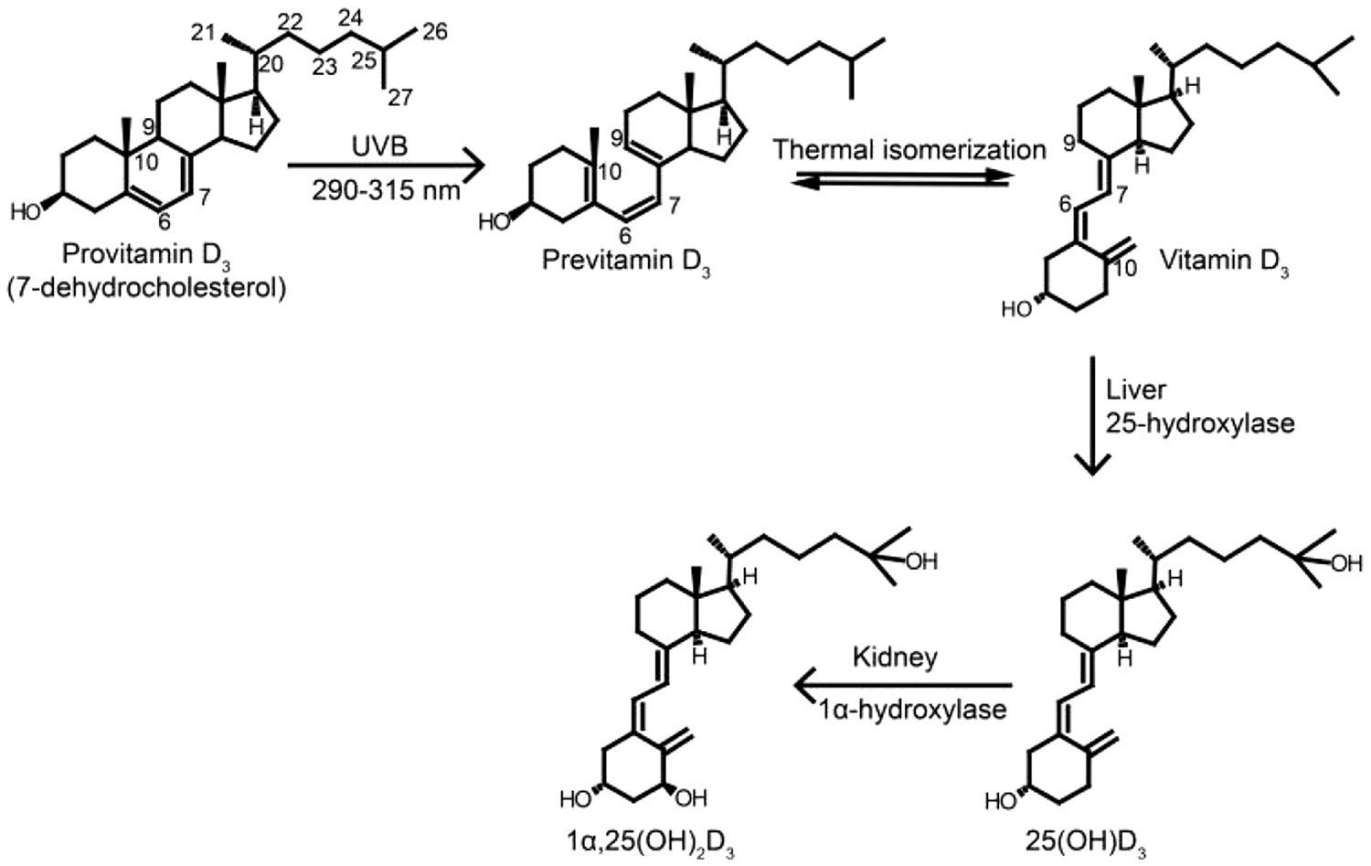
Chemical structures of Vitamin D synthesis and activation

This vitamin seems to be a prohormone that is physiologically metabolised to 1,25-dihydroxyvitamin D [1,25(OH)2D] that activates its cellular receptor (VDR or receptor of vitamin D), causing changes in the transcription levels of target genes involved in biological responses (Fig. 3).

Vitamin D may be derived from animal products like cod liver oil, salmon, tuna, beef, eggs, and chicken breast, as well as milk, yoghurt, and cheese (especially cheddar). Certain mushrooms contain vitamin D2; moreover, some commercially marketed mushrooms contain increased levels of D2 as a result of being purposefully exposed to high levels of UV radiation.

The epidermal keratinocytes are unique in that they not only synthesise vitamin D but also have the enzymatic mechanism to convert vitamin D into its active form, calcitriol (1,25(OH) (Zahoor et al. 2021). The level of epidermal pigment and the exposure intensity both correspond with the passage of time necessary to obtain this optimum previtamin D3 concentration, but they have no effect on the highest level attained. Melanin inside the epidermis can diminish the efficiency of sunlight in creating vitamin D3 within the skin by absorbing UV rays (Maurice et al. 1987; Zahoor et al. 2021) Once generated inside the skin, previtamin D3 undergoes thermal isomerization to vitamin D3. After attaching to specific binding proteins, vitamin D3 is delivered towards the liver organ, where it can be hydroxylated and then transformed into 25-OHD. Vitamin D-binding proteins transport 25-OH D to the kidney, where it is converted to 1,25(OH)2D, the hormonally active form. (Niino et al. 2015). Sunlight destroys any extra vitamin D3 or previtamin D3, therefore excessive sun exposure does not result in vitamin D3 overdose (Niino et al. 2015).

TLRs are activated by bacterial stimuli, which enhance VDR synthesis and 25-hydroxyvitamin D-1-hydroxylase activity. Cathelicidins seem to be proteins that bind to and kill bacteria. Several studies, on the other hand, found that vitamin D and VDR had an effect on both B and T cells, as well as the adaptive immune response (El-domyati et al. 2007). However, vitamin D might alter B-cell activity by limiting differentiation and proliferation, inducing apoptosis, and, lastly, lowering immunoglobulin synthesis, including autoantibodies. Vitamin D may potentially alter T-cell activity by lowering T helper (Th) cell proliferation as well as differentiation and supporting a transition from a pro-inflammatory into a more tolerogenic immunological condition.

Vitamin D has been discovered in research to inhibit the cytokine production required for Th17 and Th1 differentiation, encourage T cells to release IL-10, which is an anti-inflammatory Th2 cytokine, and thus decrease the expression of cytokines such as TNF- and IL-8, IL-2 interferon-y, and decrease the intensity of class II molecules of the main histocompatibility complex that affect dendritic cells (Islands 2005). Such substantial impacts on T-cell growth and cytokine expression contribute to the processes behind vitamin D's therapeutic activity on psoriatic skin. The characteristic clinical signs of erythematous scaling plaques in psoriatic skin are, as previously stated, the outcome of keratinocyte hyperproliferation and aberrant differentiation. The hyperproliferative condition is distinguished by a rising prevalence of proliferating basal cells, a shorter keratinocyte cell cycle (36 h against 311 h in normal skin), and a reduced epidermal turnover period (4 days from the basal cell layer to the stratum corneum, versus 28 days in normal tissue) (Trémezaygues and Reichrath 2011).

The abnormal differentiation of the psoriatic area is more severe, as indicated by a postponement in the production of K10 and K1, which are found in healthy skin, as well as K16 and K6 overexpression, which is seen in repairing skin. Vitamin D promotes keratinocyte development and growth and guards them against premature death at physiological concentrations; however, at a pharmaceutical (106 m) dose, vitamin D suppresses the proliferation of keratinocytes and has a specific proapoptotic impact (Bernengo 1998).

These impacts on keratinocyte development and proliferation are critical to vitamin D's function in the treatment of psoriasis. Additionally, as previously stated, vitamin D regulates the production of K10 and K10 inside the spinosum stratum, those essential keratins whose expression is postponed in psoriatic skin (Zahoor et al. 2021).

Moreover, vitamin D has already been demonstrated to restore the dispersion of integrins, including ICAM-1, HLA-DR, and CD26, along the dermal-epidermal interface, which is disrupted in lesional skin (Garbicz et al. 2022). It is theorised that the different location of integrins causes keratinocyte overproliferation and loss of adhesive ability in psoriasis (Garbicz et al. 2022). The impact of vitamin D topical therapies on integrin structures in non-lesional, lesional psoriatic, as well as regular-skinned patients was investigated. The staining patterns of the integrin subunit were normalised in lesional psoriatic skin after treatment, but not in non-lesional psoriatic skin before and after treatment. (Meydani et al. 2018).

### Vitamin E

Vitamin E, which is lipid-soluble, is a powerful antioxidant found in all cell membranes. Vitamin E exists in eight forms in nature (-, -, -, -tocopherols, and also -, -, -tocotrienols), although -tocopherol is the most physiologically active (Ji and Liu 2019). An investigation found a statistically significant drop in vitamin E plasma levels in twenty psoriatic patients when compared to normal individuals. Patients with the most severe illnesses had the lowest vitamin E plasma levels (“^48^.pdf”. 2022). Vitamin E levels in the blood of seven individuals with psoriatic acral pustulosis or erythrodermic psoriasis, whether or not linked with persistent drinking, were also tested throughout and after the acute stage. Only in individuals with a history of alcohol addiction (*n* = 5) was vitamin E lowered to levels below the range of normal during the severe psoriatic phase, indicating that vitamin E insufficiency should be explored when pustular psoriasis or erythrodermic is coupled with chronic drinking (Paper 2013a). A new randomised case–control study of 60 patients with autoimmune skin disorders looked at vitamin E levels in tissue and sera. Psoriasis, alopecia areata, and vitiligo patients showed lower tissue and blood vitamin E concentrations than healthy controls (*n* = 15 in each group, *p* 0.001). There exists a relationship between the pathophysiology of several autoimmune illnesses and oxidative stress, suggesting that antioxidant medicines may play a role in their therapy (Fairris et al. 1989).

Many investigators reported that the antioxidant combination of vitamin E, selenium, and coenzyme Q (10) was introduced to the diets of individuals having severe psoriasis to evaluate its influence on disease progression (Miroddi et al. 2015). In this randomised, double-blind clinical research, twenty-eight cases of severe psoriatic arthritis (PsA) and thirty individuals with erythrodermic psoriasis (EP) were included. EP and PsA patients were assigned at random to either a treatment (antioxidant supplements plus conventional therapy) or control subjects (placebo plus conventional therapy). Throughout the trial period, clinical advancement in the supplemented groups was substantially faster than in the control groups. The patient group treated with conventional medication plus antioxidant supplements had considerably decreased illness severity at 30 days, as measured by subjective grading. The antioxidant mix included 50 mg/day of Coenzyme Q(10) (ubiquinone acetate), 50 mg/day of natural vitamin E (-tocopherol), and 48 mg/day of selenium (aspartate salt).

### Aloe vera

*Aloe barbadensis* Miller (also called *Aloe vera* Linnaeus) seems to be a succulent tropical plant of the Liliaceae family. Aloe leaf pulp contains 98.5% water, while gel or mucilage contains 99.5% water. Carbohydrates, proteins, mucopolysaccharides, enzymes, anthraquinones, salicylic acid, chromones, vitamins, and minerals also make up the remaining 0.5–1%. (Aghmiuni 2017). Acemannan and Aloe-emodin are antibacterial active ingredients that can help with psoriasis. Furthermore, owing to its keratolytic activity, salicylic acid eliminates psoriatic plaques. Aloe vera gel is employed to treat psoriasis by reducing redness and scaling. It is employed to make topical creams and lotions. (Choonhakarn et al. 2010). Aloe vera also has immunomodulatory, antioxidant, anti-inflammatory, anti-fungal, and anti-tumor properties. It also promotes skin hydration and wound healing by increasing collagen activity. This aids in the healing of the psoriatic skin's negative consequences (Choonhakarn et al. 2010).

In a randomised study of 80 individuals with mild and moderate psoriasis vulgaris, Aloe vera and triamcinolone acetonide (TA, 0.1%) were compared. The average Severity Index of Psoriasis Area (PASI) value decreased by 7.7 in the group using aloe vera and 6.6 in the group using TA after eight weeks of therapy. The TA group's average DLQI (Index of Dermatology Life Quality) declined by 5.8 points, while the Aloe Vera group's decreased by 6.1 points. It was discovered that aloe vera cream was much more effective (Dhanabal et al. 2011). Extracted Aloe vera had 81.95% anti-psoriatic action in micetail models, compared to 87.94% for tazarotene (Divya et al. 2016). Divya et al. created a local nanogel with aloe-emodin (an anthraquinone found in Aloe vera) and acitretin using chitin. Their blood compatibility was demonstrated in vitro. Investigations in Perry's mouse tail as well as skin safety trials showed the formulation's potential effectiveness in psoriasis therapy (Rajiv et al. 2019).

### Angelica sinensis

*Angelica sinensis* (Dong Qquai, female ginseng) seems to be a biennial or perennial plant in the family Apiaceae. It is also known as Dong Quy. Something has long been utilised in traditional Chinese medicine (TCM). TCM claims that it stimulates and replenishes blood while also correcting insufficiency. Psoralen, a powerful furocoumarin, is found in Angelica sinensis extract. Psoralens are used in the treatment of psoriasis as they behave as photosensitizers when exposed to UV-A. Patients self-administer PUVA therapy by eating Angelica sinens and then exposing themselves to natural sunlight or UV. After consuming Angelica sinens, UV exposure promotes DNA cross-linking in the epidermis, slowing the frequency of epidermal DNA synthesis (Richard 2020). Furthermore, they produce mitochondrial malfunction, Langerhans cell toxicity, reactive oxygen species formation, and keratinocyte and lymphocyte death (Sivanesan et al. 2009). In a randomised, double-blind study, the PASI score was used to assess the efficacy of psoralen orally plus UV-A in the treatment of plaque psoriasis. After twelve weeks of therapy, two-thirds of patients improved their PASI score by at least 75%, compared to 0% in the placebo plus UV-A group (Kerkhof et al. 2006).

### Araroba tree (*Vataireopsis araroba* (Aguiar) Ducke)

Dithranol (synonym: anthralin), an anthracene derivative, is an effective topical therapy for psoriasis. It was derived from chrysarobin, which was taken from the araroba tree's bark, which thrives in the Amazon rain forests. Dithranol suppresses the production of pro-inflammatory cytokines as well as keratinocyte growth. A randomised study on 106 patients with severe psoriasis lesions found that 15:45 min of dithranol quick contact treatment with stepwise increases in dithranol concentration up to 5% once every day for twelve weeks were significantly more effective than the standard care with 50 g/g of calcipotriol ointment twice every day. (Moy et al. 2018).

### Barberry bark (*Mahonia aquifolium* (Pursh) Nutt.)

*Mahonia aquifolium*, sometimes called Oregon grape, is just an evergreen shrub in the Berberidaceae family. It is indigenous to the United States and is employed to treat a variety of inflammatory cutaneous conditions. It has long been used in TCM. The healing effect of Mahonia aquifolium versus psoriasis is due to berberine, an alkaloid found in the plant's extract. Berberine has been demonstrated to have anti-inflammatory effects through a variety of mechanisms, such as the downregulation of lipoxygenase as well as lipid peroxidation, a decrease in the infiltration of T cells in exposure lesions, a decrease in cyclooxygenase activity, resulting in a reduction in the suppression of IL-8, and prostaglandin E2. Berberine suppresses cell development by intercalating into DNA, preventing replication of DNA and cell proliferation. Other alkaloids that inhibit lipoxygenase, such as oxyberberine, jatrorrhizine, corytuberine, and columbamine, contribute to anti-inflammatory action. (Wiesenauer and Lootke 1996). An individual study was carried out to assess the performance of an ointment comprising 10% Mahonia aquifolium bark extract. It was thought to be beneficial in treating moderately severe psoriasis vulgaris (Kost et al. 2001). The Mahonia aquifolium crude extract suppressed IL-8 production, which is significant in the treatment of psoriasis. The crude extract's main components were bisbenzylisoquinoline alkaloids and protoberberine. The first group inhibited the production of IL-1, T cells, TNF-, and TNF- (Ghazisaeedi et al. 2022).

### Basil

The activated version of NF-B has been linked to osteoporosis, psoriasis, septic shock, AIDS, and other inflammatory illnesses (Cyclin 2003). It has previously been reported that "holy basil" possesses chemopreventive properties. Ursolic acid, a tri-terpenoid produced from basil and rosemary, has been demonstrated to limit NF-B activation by inhibiting IKK, which in turn suppresses cyclin D1, COX-2, and matrix metalloproteinase-9 (Woolf 2018).

### Capsicum annum

*Capsicum annuum* is considered a plant species endemic to southern North America, northern South America, and the Caribbean. Cultivars derived from the native American bird peppers can still be found in the Americas' warmer areas. Some woody types of this species were previously referred to as C. frutescens; however, the characteristics used to differentiate those types are seen in numerous species like C. annuum, but they are not consistently recognised in C. frutescens types. Numerous chemicals, including neuropeptide Y, protein gene product 9.5 (PGP-9.5), nerve growth factor (NGF), CGRP, and SP, have been linked to itchy skin in psoriasis. NGF, PGP 9.5-reactive nerve fibers, and the number of NGF-immunoreactive keratinocytes have been found to be elevated in itch skin and related to itch intensity. TrkA, an NGF high-affinity receptor, is abundantly expressed in the epidermal and dermal nerve fibres, and this corresponds with itch intensity (Henrich et al. 2015). Keratinocytes and nerve fibres in itchy psoriatic skin have higher levels of SP and its receptor expression. Furthermore, capsaicin was demonstrated to considerably decrease itch in psoriasis patients, indicating a potential role for this neuropeptide in the aetiology of psoriasis itching (Znajdek-awi 1124).

### *Centella asiatica* L.

Guto Kola, Hydrocotyle asiatica L, or Centella asiatica is commonly used in dermatology to treat skin problems.It is a member of the Apiaceae family. The primary ingredients of *Centella asiatica* are centelloids, as these represent pentacyclic triterpenoids that include madecassoside, asiaticoside, madecassic acid, and asiatic acid. It also includes saponins of the oleanane and isothankunic acid types, such as terminolic acid and centellasaponin D. It has saponins (1–8%) and essential oils (1%) (Sampson et al. 2001).

Centella asiatica has long been used in Ayurvedic medicine to treat a variety of diseases. It was discovered to have tremendous promise as a natural antioxidant and a DNA damage preventer. A research study compared the madecassoside and asiaticoside anti-psoriatic actions on the development of keratinocytes (SVK-14) with dithranol and psoralen. The madecassoside and Asiaticoside IC50 values were discovered to be 8.6 0.1 M and 8.4 0.6, respectively. In addition, the aqueous product of the herb Centella asiatica was shown to be less effective than Psoralea corylifolia seeds. However, the findings were equivalent to dithranol's IC50 value of 5.2 0.4 M (Ouyang et al. 2016).

Madecassoside ointment has been employed in research to examine the impact of madecassoside against imiquimod-induced psoriasis-like dermatitis using BALB/c mice. Il-17 and IL-23 are important in the development of psoriasis. According to real-time PCR results, madecassoside significantly reduced mRNA levels of IL-23 and IL-17. Furthermore, keratinocyte proliferation was reduced using haematoxylin, 5-bromo-2'-deoxyuridine (BrdU), and eosin staining incorporation experiments. The number of Th17 cells was shown to be lower using flow cytometry. Consequently, madecassoside ointment was discovered to be beneficial in the treatment of psoriasis via the IL-17 and IL-23 axes (Genoux and Duffy 2019).

### Cestrum diurnum

*Cestrum diurnum*, sometimes termed "wild jasmine," is a member of the family Solanaceae and represents a West Indian plant widely grown as a decorative plant. The plant's leaves contain a strong steroid glycoside that mimics vitamin D action. It includes 1,25-dihydroxycholecalciferol glycosides. Vitamin D3 is normally generated at the skin via UV light-dependent processes or obtained through food. To produce the most activated vitamin D3, 1,25-dihydroxycholecalciferol, vitamin D3 is hydroxylated twice, in the liver and by the kidney (calcitriol) (Prema and Raghuramulu 1994). Topical vitamin D has a therapeutic impact via two mechanisms: the vitamin D receptor-mediated method, which inhibits keratinocyte growth, and the non-genomic mechanism, which stimulates keratinocyte differentiation due to elevated intracellular calcium. As a result, it has now become a localised agent in the treatment of psoriasis.

Research was done to explore the effect of its supplementation on psoriasis. For six months, patients were given vitamin D2 only once every 2 weeks. Both the psoriasis area and the PASI improved after 3 and 6 months. The average PASI improvement was 34.21% versus 1.85% for the placebo. Vitamin D supplementation increased the results of psoriasis therapy (Das et al. 2022). Aurochem Laboratories Pvt. Ltd. markets Cestrum diurnum extract (3 g/g) as an ointment and gel under the brand name PsoriaBan Natural. It has shown up to 89% effectiveness. It has been proven to be helpful in the healing of psoriasis in the face and scalp.

### Cloves

Cloves' anti-inflammatory and antioxidant properties are well documented. The active ingredients in cloves are eugenol and isoeugenol. Several studies have demonstrated that these chemicals can inhibit NF-B activation by inhibiting IB breakdown (Murakami et al. 2003). Murakami et al. (Barrea et al. 2018). found that bis-eugenol, but not eugenol, inhibits IB degradation and suppresses inflammatory cytokine production at both the gene and protein levels.

### Coffee

Coffee is considered one of the most commonly drunk drinks, regardless of location. According to data (Manuscript 2017), only tea and water are more commonly consumed liquids. Coffee, furthermore, is a pharmacologically active fluid. Its composition contains several physiologically active chemicals (Gokcen and Sanlİer 2017). This would include lipids, carbohydrates, nitrogenous chemicals, vitamins, minerals, antioxidants, phenolics, lactones, alkaloid compounds, diterpenes, and caffeine, which accounts for around 1% of all coffee composition. This compound has a variety of medicinal properties and has been shown to inhibit monocyte and neutrophil migration, lower levels of glucose in the blood, have immunosuppressive and anti-inflammatory properties and safeguard from neurodegeneration (Sharif et al. 2017).

Caffeine is the most researched component in coffee. It inhibits Th1/Th2 cell proliferation, as well as the production of pro-inflammatory cytokines like TNF-, IL-1, IL-6, and IL-11, while simultaneously releasing anti-inflammatory markers like adiponectin, IL-4, and IL-10 (Hall et al. 2015). Furthermore, it inhibits cyclin Adenosine Monophosphate (cAMP), which acts as an immunomodulator, increases the production of anti-inflammatory cytokines, and acts as an antagonist of the adenosine receptor. Moreover, the presence of polyphenols in coffee's composition contributes to its anti-inflammatory properties. A subset of these chemicals, particularly chlorogenic acid and its metabolites block pro-inflammatory cytokines, whereas caffeic acid lowers levels of nitrite and eliminates inflammatory symptoms (Zampelas et al. 2018).

According to Zampelas et al. (Fernandez et al. 2012), coffee drinking boosts the body's inflammatory process, which could have a negative correlation with psoriasis severity. According to this study, drinking coffee on a daily basis increased TNF-, CRP, and IL-6 levels, which resulted in severe pathological manifestations of psoriasis. It is crucial to note, however, that this study linked heavy coffee intake (more than 200 mg per day) to psoriasis severity. The hazard of psoriasis was shown to be modestly linked with an elevation in coffee intake, albeit this was not significant statistically among cigarette consumers. It is worth noting that this study may have had a significant limitation: a variety of caffeine sources, such as sweetened beverages and overly processed meals, were tested, which may have influenced the outcomes reached. According to Sharif et al. (Hall et al. 2015), regular coffee consumption increases the level of anti-inflammatory elements while decreasing the synthesis of pro-inflammatory factors (particularly TNF-), which is important in reducing the severity of psoriasis. Several studies have found that the effect of coffee varies depending on the dose. Moderate-intensity coffee consumption up to three cups per day reduces psoriasis manifestations and has an anti-inflammatory impact, whereas greater coffee consumption, particularly more than four cups per day, worsens clinical psoriasis symptoms and is related to a rise in pro-inflammatory chemicals (Ammon and Wahi 1990).

### Curcumin

Curcumin is indeed a polyphenol produced from the yellow spice turmeric, a member of the Zingiberaceae family, with several characteristics. It contains anti-tumor, anti-oxidant, anti-inflammatory, and many other biological properties and is chemically identified as [1,7-bis(4-hydroxy-3-methoxyphenyl)-1, 6-heptadiene-3, 5-dione] (Editor 2017). It's apparent that curcumin's widespread usage in medicine is due to its various qualities, which include anti-inflammatory, antioxidant, anti-proliferative, anti-microbial, and anti-carcinogenic capabilities (Gupta et al. 2011). Curcumin is used to treat a variety of ailments, including rheumatoid arthritis, eye problems (such as uveitis anterior chronica and conjunctivitis), menstrual irregularities, infections in the urinary tract, and gastrointestinal and liver issues (e.g., irritable bowel disease, abdominal pain). It's also utilised as an adjuvant treatment for cancer of the skin, wound repair, and chicken pox (Anand et al. 2007). Dendritic cells have been linked to the early stages of psoriasis. Myeloid dendritic cells secrete IL-23 and IL-12, which stimulate IL-17-producing Th22, Th1, and T cells, resulting in the production of inflammatory cytokines such as IFN-, IL-17, IL-22, and TNF, which trigger the cascade associated with psoriasis inflammation (Skyvalidas 2020).

Curcumin contains anti-oxidative, anti-inflammatory, and immunomodulatory properties and can decrease pro-inflammatory factors, activation of T cells, and proliferation via working on the AP-1, NF-B, and MAPK pathways. It can keep DC immature, which affects cytokine generation, stimulation of responsive T cells, and antigen presentation. Curcumin inhibits the production of IL-17 by CD4+ T cells (Campbell, et al. 2018). Curcumin inhibits the development of imiquimod-induced differentiated HaCaT cells by downregulating pro-inflammatory cytokines TNF-, IL-17, and IFN- (Kang et al. 2016). IFN- and TNF-, as well as IL-23, IL-22, IL-12, and IL-2, returned to normal levels in mice after curcumin administration. This might be because curcumin reduces Kv1.3 channel currents, inhibiting T cell proliferation, or because curcumin influences AP-1, NF-B, and MAPK signalling pathways inside psoriasis mice (He et al. 2015). In an imiquimod-stimulated psoriatic model, curcumin nanohydrogel restored the normally distributed TJs proteins ZO1 and occludin while decreasing iNOS and TNF- production (Zhang, et al. 2019). Following topical application to mice, curcumin reduced inflammatory symptoms, mRNA levels of IL-1, IL-22, IL-17F, IL-17A, and TNF-, and protein expression of CC Chemokine receptor 6 (CCR6) (Kurd et al. 2008).

### Fennel and anise

As estrogenic agents, fennel (*Foeniculum vulgare*) and anise (*Pimpinella anisum*) have been used. They are said to enhance milk supply, induce menstruation, assist delivery, decrease male climacteric symptoms, and boost libido. The major ingredient of anise essential oils and fennel, anethol, was thought to be a powerful estrogenic agent, but subsequent research reveals that the true pharmacologically active molecules are anethol polymers, including dianethol and photoanethol (Sen et al. 1996). Anethole has been demonstrated to inhibit inflammation as well as carcinogenesis. It has been demonstrated to have antioxidant properties. We demonstrated that anethole can limit NF-B activation by inhibiting IB breakdown (Yilmaz et al. 2010).

### Garlic

Garlic (*Allium sativum*) is a well-researched, great herbal remedy that has been utilised for millennia to cure a variety of health issues (Pazyar and Feily 2011). Its contents include sulfur-containing molecules such as alliin, enzymes such as alliinase, and chemicals enzymatically synthesised from alliin as allicin. Garlic also contains other elements such as oligosaccharides, flavonoids, arginine, and selenium (Allison et al. 2006). One complicated combination is aged garlic extract (AGE). Allin, cyclophilin, *S*-methyl-l-cysteine, *S*-allyl-l-cysteine, *S*-acetylcysteine, *S*-allylmercapto-l-cysteine, *S*-1 propionyl-l-cysteine, fructose-arginine, and beta-chlorogenic are among its constituents. l-Arginine, l-methionine, and l-cysteine are also present. Psoriasis is now associated with the activity of the nuclear transcription factor kappaB. Extensive investigation has revealed this path. Garlic (*S*-allyl mercapto cysteine, diallyl sulfide, ajoene) can inhibit this transcription factor (Singh and Tripathy 2014).

### *Gaultheria procumbens* L.

*Gaultheria procumbens* (eastern teaberry, boxberry, checkerberry) seems to be an herbaceous plant that yields essential oils and belongs to the Ericaceae family. These oils include the anti-inflammatory compound methyl salicylate (Jurek and Olszewska 2019). The salicylate, as well as the procyanidin-rich stem extract from Gaultheria procumbens, suppresses pro-inflammatory proteins such as hyaluronidase, COX-2, and lipoxygenase. Models created in vitro showed antioxidant effects. Ex vivo experiments in human neutrophils stimulated with *N*-formyl-l-methionyl-l-leucyl-l-phenylalanine and lipopolysaccharides reduced the production of cytokines and proteinases, as well as reactive oxygen species (Saha et al. 2014).

### Ginger

Ginger (*Zingiber officinale*) would be a blooming medicinal plant with a spicy root or rhizome (plant stem) (Salafzoon 2017). Furthermore, it is extensively utilised in folk medicine due to its numerous health advantages in a variety of conditions, covering chronic conditions such as diabetes (Wang et al. 2011), cancer (Papers 2013b), ulcers (Kukula-koch et al. 2018), Alzheimer's disease (Masuda et al. 2004), cardiovascular disease (Al et al. 1449), and depression (Rabelo et al. 2014). Ginger's positive effect on these disorders is mostly due to its antioxidant (Pkurmaceutical 1933) and anti-inflammatory characteristics (Tjendraputra et al. 2001).

The sharp scent and flavour of new ginger root are caused by active volatile oils (e.g., shogaols, gingerols, and zingerone), which account for around 1–3% of its weight (Article 2001). Ginger also includes antioxidants like vitamin C, vitamin E, lutein, beta-carotene, lycopene, quercetin, genistein, and tannin (Wagesho and Chandravanshi 2015). In addition, ginger includes important nutrients such as manganese, selenium, copper, and zinc (Wagesho and Chandravanshi 2015).

Gingerol, the active component, has already been demonstrated to have chemopreventive properties (Tjendraputra et al. 2001). Gingerol also suppresses the nitric oxide synthase enzyme and cyclooxygenase (COX-2), both of which are recognised to be NF-B regulated (Article 2001). Because gingerol may reduce platelet aggregation, synthesised gingerol analogues with increased potency as platelet aggregation inhibitors, similar to aspirin, may be useful in cardiovascular disease (Getaneh et al. 2021). Since ginger contains anti-inflammatory effects, it is a potential herbal medicine for the treatment of psoriasis.

### Indigo (*Baphicacanthus cusia*, Brem.)

TCM considers "Indigo naturalis" to be an important medication. It's a blue powder obtained by grinding the *Baphicacanthus cusia* plants, fermenting them, and adding lime to them. Forty-two people with chronic plaque psoriasis were given ointment containing 10% indigo once a day for 12 weeks in a randomised placebo-controlled experiment. The used indigo naturalis includes 1.4% indigo and 0.16% indirubin. The indigo therapy alleviated symptoms by 81%, whereas the placebo therapy only reduced symptoms by 26% (Lin et al. 2014). Several investigations using the indigo extract for psoriasis have been conducted. A new randomized, controlled study of 100 psoriasis patients revealed dose-dependent efficacy of indigo extract given twice daily for 8 weeks. The PASI was lowered by 50% with indigo extraction (50 g/g) and 70% with indigo extract (200 g/g). Nasopharyngitis, infections of the upper respiratory tract, and local erythema were reported in certain individuals. There were no severe negative effects noted (Markham et al. 2003). Before and after therapy, punch biopsies revealed normalisation of epidermal appearance as well as a decrease in the main pro-inflammatory cytokine in psoriasis (Markham et al. 2003).

### Lace flower (*Ammi visnaga* (L.) and *Ammi majus* (L.))

5-Methoxypsoralen (5-MOP) and furanocoumarins 8-methoxypsoralen (8-MOP) is secluded for therapeutic application from *Ammi visnaga* (L.) Lam. and *Ammi majus* (L.) Psoralens are phototoxic chemicals that are photoactivated via ultraviolet A (UVA) light and can induce extremely phototoxic skin responses.

They limit keratinocyte growth and exhibit immunosuppressive effects in the healing environment of PUVA treatment (psoralen and UVA), which is utilized to treat strong inflammatory skin disorders such as psoriasis. Several clinical investigations have demonstrated the effectiveness of systemic PUVA (Vongthongsri et al. 2006), bath PUVA (Amornpinyokeit and Asawanonda 2006), and cream PUVA (0.1% 8-MO) (Bensouilah and Bensouilah 2003) in psoriasis.

### *Matricaria recutita* L.

*Matricaria chamomile*, or *Matricaria recutita*, is a part of the Asteraceae family. It has traditionally been employed to alleviate digestive issues. Chamazulene seems to be the primary phytochemical accountable for antipsoriatic action. It's a derivative of the oil extraction matrix derived from flowers (Rauf 2021). Chamazulene exerts anti-inflammatory action by suppressing lipoxygenase, which inhibits leukotriene B4 synthesis (LTB4). LTB4 production then increases, resulting in psoriatic plaque. Therefore, Chamazulene's positive impact will be demonstrated by inhibiting LTB4. The flavonoids and apigenin The flower contains quercetin (Bonesi et al. 2018).

Quercetin is an anti-inflammatory, anti-tumor, antiviral, and antibacterial flavonol. It inhibits both IFN-induced STAT-1 stimulation and NF– activation. It reduces the generation of histamine and IgE. It inhibits the enzymes nitric oxide synthase (iNOS), TNF-, and IL. Quercetin works through many processes and might be used to treat psoriasis. Apigenin is indeed a flavone with anti-inflammatory and antioxidant properties. It inhibits NF-B activation and decreases TNF-induced luciferase reporter gene transactivation (Jamalian et al. 2012). It also lowers TNF-, suppresses COX-2, IL-6, and IL-8 expression, and inhibits TNF–induced luciferase reporter gene transactivation (Jamalian et al. 2012). It also protects against fungal infections (Silva et al. 2019). Chamomile essential oil inhibits NF-, TNF-, IL-1, IL-6, iNOS, and COX-2 (Gad et al. 2019).

### Melaleuca alternifolia

The Melaleuca plant is endemic to Oceania and is utilised in traditional medicine in Australia. -terpinene, -terpineol, terpinen-4-ol, 1,8-cineol, -pinene, terpinolene, sabinene, and limonene are all found in tea plant oil. Sesquiterpenes and aromatic chemicals make up the remainder of the oil. In vivo, tea tree oil also has substantial antibacterial and antifungal properties. Methyl eugenol, terpinen-4-ol, and 1,8-cineol all play important roles in antibacterial action (Pazyar and Yaghoobi 2012). Terpinen-4-ol has been shown to reduce TNF-, IL-1, IL-8, and PGE2 production. It can also influence vasodilation and plasma extravasation (Koh et al. 2002). In a trial of 27 patients to assess the impact of tea oil on weeping or flare induced by histamine, the volume of mean weeping decreased significantly after 10 min of treatment (Khalil et al. 2004). Human and rodent investigations had similar findings. Tea plant oil reduced the susceptibility to flared and wheal histamines. Tea tree oil, on the other hand, has been linked to contact allergies in a small number of people. Freshly extracted oil is a mild to the moderate sensitizer, while oxidation elevates the oil's allergenic action. The oil contains sensitizers such as -terpinene, ascaridole, -phellandrene, -phellandrene, limonene, and trihydroxymenthane. The majority of the responses are caused by direct tea tree oil application (Ali and Blunden 2002), Tea tree oil taken orally can ultimately cause systemic contact dermatitis, cognitive confusion, and coma (Jurek and Olszewska 2019).

### Nigella sativa

*Nigella sativa* is a popular therapeutic plant that has a long religious and cultural background in Unani, Ayurvedic, Arabic, and Chinese medicine. Many bioactive natural agents, including alpha-hederin, alkaloids, thymoquinone, and saponins, are found in N. sativa and contribute to its wide spectrum of effects as a bronchodilator, diuretic, antidiabetic, analgesic, antihypertensive, antibacterial, antineoplastic, and anti-inflammatory agent, making it a promising therapeutic for dermatological diseases. Thymoquinone (TQ) is the major bioactive ingredient, accounting for 30–40% of essential oils (Arjumand et al. 2019). TQ's high antioxidant and anti-inflammatory actions have prompted much investigation into its wide spectrum of health benefits. The N. sativa treatment has been proven to dramatically decrease inflammation via TQ-mediated secretion of proinflammatory cytokines as well as eosinophils (Arjumand et al. 2019). Several researchers investigated the impact of TQ on attenuating inflammation of the airways in a rat model of allergic asthma (El-mahdy et al. 2005). TQ intraperitoneal administration prior to airway challenge showed a significant reduction in eosinophilia in the lung, serum IgG and IgE levels, and IL-13, IL-4, and IL-5 proinflammatory Th2 cytokines. N. sativa functions as an antioxidant by enhancing free radical defence by inhibiting elastase, lipid peroxidation, and myeloperoxidase (Sethi et al. 2008). Despite downregulating transcription factors such as NF-B and STAT3 and antiapoptotic BCL2, N. sativa increases cellular death by up-regulating proapoptotic caspases 8, 9, and 3 and the B-cell lymphoma 2 associate X protein (BAX) (Forouzanfar et al. 2016). Because of its antibacterial, anticancer, anti-inflammatory, and other qualities, as well as its capacity to cure hypopigmentation through enhanced melanin distribution, N. sativa has high promise for treating a variety of dermatological diseases. N. sativa is a potential treatment for plaque psoriasis since it has few side effects and is widely available.

### Olibanum (*Boswellia serrata*, Triana & Planch.)

Frankincense, also known as olibanum, is a yellowish-brown oleo-gum resin extracted from Boswellia species such as *Boswellia serrata* (Woolley et al. 2012). The genus Boswellia has around 25 species (Balamurugan et al. 2022). Several previous studies reported that 200 patients with mild to moderate psoriasis were given an olibanum ointment containing 5% 3-*O*-Acetyl-11-keto-boswellic acid three times per day for twelve weeks. The PASI, as well as other blood indicators including leukotriene B4, TNF, VEGF, and PGE2, were dramatically decreased. Contact dermatitis occurred in 13 individuals (6.5%) (Balamurugan et al. 2022).

### Pomegrante

Dried pomegranate, or Punica granatum, seeds are a popular culinary spice. Pomegranate extract flavonoids have been shown in atherosclerotic humans and mice to reduce the oxidation of low-density lipoprotein and cardiovascular disease (Aviram and Dornfeld 2001). Pomegranate juice is known to suppress serum angiotensin-converting enzyme activity and lower systolic blood pressure (Schubert et al. 2002). Pomegranate can reduce NF-B activation in vascular endothelium via a unique method, according to new research (Asogwa and Okoye 2019).

## Conclusion

From this review, it has become clear that naturally derived compounds could very likely become key players in future psoriasis treatments. This article has summarised some of the compounds and plants that have been studied to date for their possible anti-psoriasis properties. Many more untapped resources, however, remain in nature. Phytochemicals have been reported to possess numerous health benefits, and ongoing research is being conducted to determine their physiological effects. The traditional use of natural compounds in psoriasis treatment is relatively cheap due to the availability of plants and the simple methods used in product preparation. However, the commercialization of natural compounds for psoriasis treatment may result in the dwindling of natural resources and problems with producing a consistent quality of adulteration. However, the use of synthetic medications has led to a variety of adverse effects, one of which is psoriasis. In recent years, a study in this sector has provided a variety of treatment options for treating psoriasis. The use of herbal medicine is one such expanding strategy. Several herbs have been demonstrated to have anti-psoriatic capabilities, as discussed within this review. Herbal treatment, on the other hand, is currently limited to a restricted subset of psoriasis patients. Herbal extracts may be used in conjunction with synthetic medications to treat psoriasis. The dual use may lead to a lower synthetic drug dosage as well as a recurrence of the negative effects. To guarantee the safety and effectiveness of psoriasis treatment, the standardisation of herbal medicines necessitates regulatory requirements and quality control processes. To assess the therapeutic efficiency of these natural chemicals, more studies with a larger sample size are necessary. Fresh plant resources should also be examined in this region.

## Data Availability

A standardized approach of herbal medicine, is needed to design larger trials which can ensure the effectiveness of the treatment of psoriasis.Moreover, Further studies using newer herpal plant are essential to establish the efficacy of these natural sources.
